# Strain and process development for poly(3HB-*co*-3HP) fermentation by engineered *Shimwellia blattae* from glycerol

**DOI:** 10.1186/s13568-015-0105-8

**Published:** 2015-03-04

**Authors:** Shunsuke Sato, Björn Andreeßen, Alexander Steinbüchel

**Affiliations:** Institut für Molekular Mikrobiologie und Biotechnologie, Westfälische Wilhelms-Universität Münster, Corrensstraße 3, D-48149 Münster, Germany; Environmental Sciences Department, Faculty of Meteorogy, Environment and Arid Land Agriculture, King Abdulaziz University, Jeddah, Saudi-Arabia

**Keywords:** Copolymerization ratio, Fermentation condition, Glycerol, Poly(3HB-*co*-3HP), *Shimwellia blattae*

## Abstract

Poly(3-hydroxybytyrate-*co*-3-hydroxypropionate), poly(3HB-*co*-3HP), is a possible alternative to synthetic polymers such as polypropylene, polystyrene and polyethylene due to its low crystallinity and fragility. We already reported that recombinant strains of *Shimwellia blattae* expressing 1,3-propanediol dehydrogenase DhaT as well as aldehyde dehydrogenase AldD of *Pseudomonas putida* KT2442, propionate-CoA transferase Pct of *Clostridium propionicum* X2 and PHA synthase PhaC1 of *Ralstonia eutropha* H16 are able to accumulate up to 14.5% (wt_PHA_/wt_CDW_) of poly(3-hydroxypropionate), poly(3HP), homopolymer from glycerol as a sole carbon source (Appl Microbiol Biotechnol 98:7409-7422, 2014a). However, the cell density was rather low. In this study, we optimized the medium aiming at a more efficient PHA synthesis, and we engineered a *S. blattae* strain accumulating poly(3HB-*co*-3HP) with varying contents of the constituent 3-hydroxypropionate (3HP) depending on the cultivation conditions. Consequently, 7.12, 0.77 and 0.32 g_PHA_/L of poly(3HB-*co*-3HP) containing 2.1, 8.3 and 18.1 mol% 3HP under anaerobic/aerobic (the first 24 hours under anaerobic condition, thereafter, aerobic condition), low aeration/agitation (the minimum stirring rate required in medium mixing and small amount of aeration) and anaerobic conditions (the minimum stirring rate required in medium mixing without aeration), respectively, were synthesized from glycerol by the genetically modified *S. blattae* ATCC33430 strains in optimized culture medium.

## Introduction

Polyhydroxyalkanoates (PHA) are polyesters synthesized by a wide range of microorganisms (Anderson et al., 1990). Most of PHA are produced from renewable resources like sugars, plant oils, glycerol, and carbon dioxide (CO_2_). As these polyesters are biodegradable, they have been expected to play an important role in environmental protection and in reduction of CO_2_ emissions, a cause of global warming (Steinbüchel and Füchtenbusch, 1998). There have been many attempts to investigate industrial production of such polymers to ascertain if they are environmently friendly or biocompatible materials (Lee, 1996; Steinbüchel, 2001). In nature, poly(3-hydroxybutyrate), poly(3HB), a homopolymer of (*R*)-3-hydroxybutyric acid (3HB), is the most abundant PHA. However, because poly(3HB) is highly crystalline, hard and brittle, its practical applications are limited. Many studies have been undertaken to improve these properties. For example, among other PHA, poly(3-hydroxybutyrate-*co*-3-hydroxyvalerate), poly(3HB-*co*-3HV), poly(3-hydroxybutyrate-*co*-3-hydroxyhexanoate), poly(3HB-*co*-3HH), and poly(3-hydroxybutyrate-*co*-3-hydroxypropionate), poly(3HB-*co*-3HP) are much more flexible and less crystalline than poly(3HB) (Andreeßen et al., 2014b; Chen et al., 2000; Doi et al., 1995; Shimamura et al., 1993; Shimamura et al., 1994). The flexibility depends on the ratio of the constituents in the copolymer. Therefore, these copolymers are accordingly expected to have a broader range of applications in packaging, agriculture and medical materials (Chen et al., 2000). Among these copolymers, poly(3HB-*co*-3HP) is considered to be very promising due to its benefiting material properties (Andreeßen and Steinbüchel, 2010).

The global glycerol production has increased rapidly during the last decade due to the increase of biodiesel production. Concomitant with the conversion of about 10 million tons of vegetable oil into biofuel, about 1 million tons of glycerol were produced as a by-product in 2011 (Quispe et al. 2013). Therefore, the aim of this study was the development of strains for poly(3HB-*co*-3HP) synthesis from glycerol as sole carbon source.

Some processes for synthesis of poly(3HB-*co*-3HP) have already been reported by Shimamura et al., 1994, Fukui et al., 2009, Wang and Inoue, 2001 and Wang et al., 2013. However, in these studies the use of expensive 3HP as precursor of 3HP-CoA (Shimamura et al., 1994; Wang and Inoue, 2001), insufficient 3HP contents to reduce the crystallinity (Fukui et al., 2009) and the requirement of high cost vitamin B_12_ are major drawbacks (Wang and Inoue, 2001; Wang et al., 2013).

Vitamin B_12_ is a cofactor of the glycerol dehydratase (Martens et al., 2002), which converts glycerol to 3-hyrdoxypropionaldehyde (3HPA), a precursor of 3HP-CoA (Wang et al., 2013), to produce 1,3-propanediol (1,3PD). However, only few bacteria are capable of synthesizing vitamin B_12_ (Sun et al., 2003). To solve this problem, we used the enteric bacterium *Shimwellia blattae* ATCC33430 (Burgess et al., 1973; Priest and Barker, 2010) which cannot naturally produce PHA but synthesizes vitamin B_12_ (Andres et al., 2004) and converts glycerol to 1,3PD.

**Figure 1:**
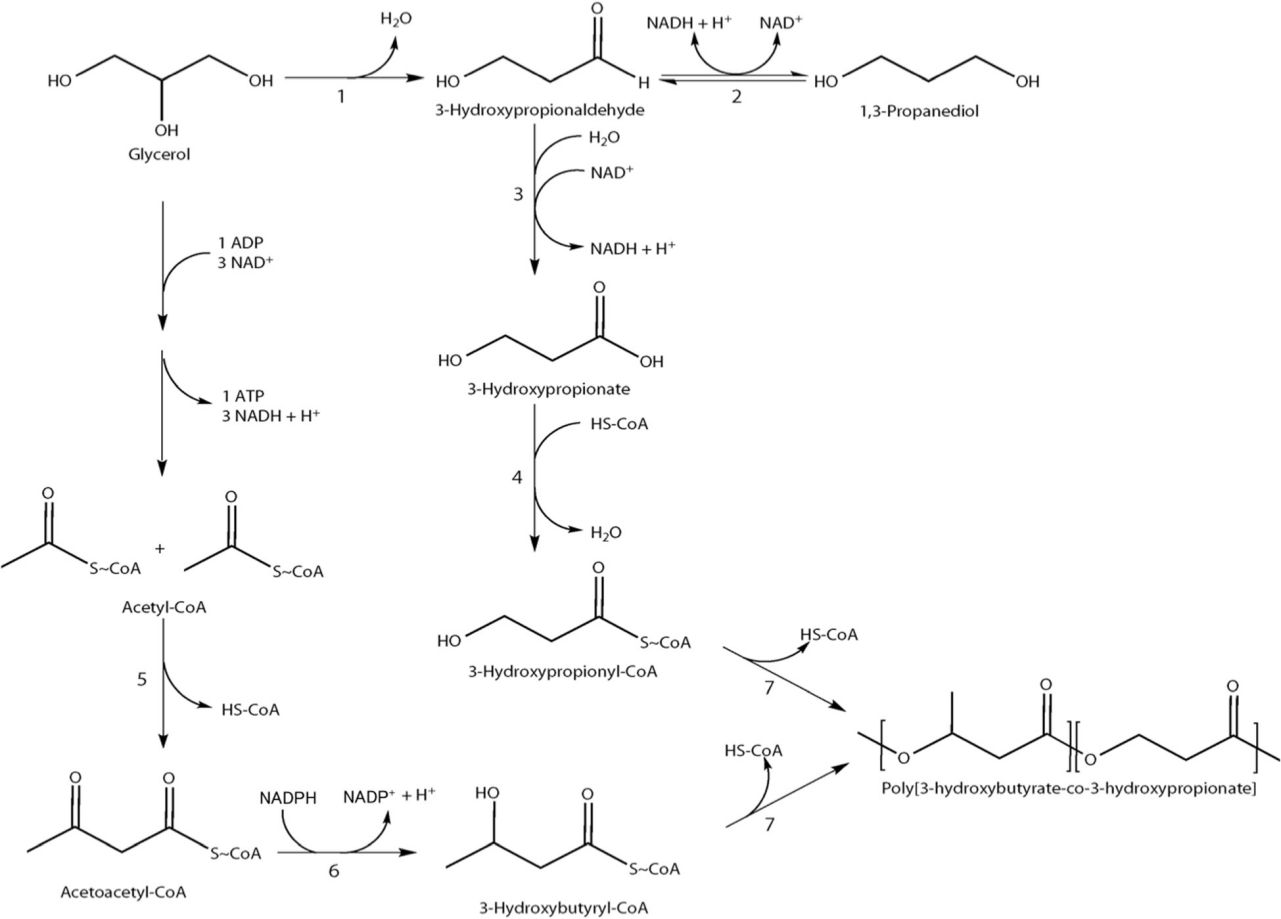
**Pathway for conversion of glycerol to poly(3-hydroxybutyrate-** ***co*** **-3-hydroxypropionate) in a recombinant strain of** ***S. blattae*** **.** 1: DhaBCE_*Sb*_, 2: DhaT_*Sb*_/DhaT_*Pp*_, 3: AldD_*Pp*_, 4: Pct_*Cp*_, 5: PhaA_*Re*_, 6: PhaB1_*Re*_, 7: PhaC1_*Re*_. Acetyl-CoA is synthesized from glycerol though glycolytic pathway.

Recently, we reported that *S. blattae* expressing 1,3-propanediol dehydrogenase (*dhaT*) and aldehyde dehydrogenase (*aldD*) of *Pseudomonas putida* KT2442, propionate-coenzyme A (propionate-CoA) transferase (*pct*) of *Clostridium propionicum* X2, and PHA synthase (*phaC1*) of *Ralstonia eutropha* H16 accumulates poly(3HP) from glycerol as a sole carbon source up to 14.5% (wt_PHA_/wt_CDW_) (Andreeßen et al., 2014a; Heinrich et al., 2013). Here, 1,3PD produced by *S. blattae* is oxidized first to 3HPA by DhaT and subsequently to 3HP by AldD. 3HP is then activated by addition of coenzyme A by Pct. In order to synthesize poly(3HB-*co*-3HP) from glycerol in *S. blattae*, we co-expressed *phaA* and *phaB1* from *R. eutropha* H16 (Budde et al., 2010) together with the enzymes for the already mentioned artificial poly(3HP) pathway (Heinrich et al., 2013). Two molecules of acetyl-CoA are condensed to acetoacetyl-CoA by a β-ketothiolase (PhaA) and acetoacetyl-CoA is then reduced by an (*R*)-specific acetoacetyl-CoA reductase (PhaB1) to generate (*R*)-3HB-CoA. As a result, the recombinant *S. blattae* (*Sb*6BP) is capable of synthesizing poly(3HB-*co*-3HP) (Figure 1).

However, the residual cell density was less than 6 g/L and therefore too low to produce much poly(3HB-*co*-3HP). Since PHA are accumulated inside the cells, low residual cell density result in only low PHA productivity. For example, even if 90 % (wt_PHA_/wt_CDW_) of poly(3HB-*co*-3HP) is accumulated in a cell, less than 54 g/L of polymer is produced under such conditions.

Therefore, an optimized culture medium was needed to overcome this problem. In this study, we report on a new strategy for synthesis of poly(3HB-*co*-3HP) using optimized cultivation medium and glycerol in genetically modified *S. blattae* without the addition of vitaminB_12_ and 3HP into the culture.

## Materials and methods

### Strain and plasmid

Table 1 lists all strains and plasmids used in this study. For cloning experiments, plasmids were transformed into *Escherichia coli* TOP10. For synthesis of poly(3HB-*co*-3HP) or poly(3HB), *S. blattae* ATCC33430 was transformed with plasmid pBBR1MCS-2::*p*_*lac*_::*aldD*:*dhaT*::*pct*::*p*_*lac*_::*phaC1AB*.

**Table 1:** **Bacterial strains and plasmids used in this study**

**Strains and plasmids**	**Relevant characteristics**	**Origin or reference**
Strains		
*E. coli*		
TOP10	*F* ^*-*^ *mcrA Δ(mrr-hsdRMS-mcrBC) φ80lacZΔM15 ΔlacX74 mupG recA1 araD139 Δ(ara-leu)7697 galE15 galK16 rpsL(StrR) endA1λ*	Life technologies (Darmstadt, D)
*S. blattae*		
ATCC33430	Wild type strain	ATCC33430
*Sb*6BP	pBBR1MCS-2 ::p*lac::aldD::dhaT:: pct::plac*::*phaC1AB* in *S. blattae* ATCC33430	This study
Plasmids		
pBBR1MCS-2	Cloning vector, Km^r^	Kovach et al., 1995
pBBR1MCS-2 ::*aldD*::*dhaT*::*pct*	Km^r^; *aldD*_*Pp*_; *dhaT*_*Pp*_; *pct*_*Cp*_	Heinrich et al., 2013
pBBR1MCS-2 ::p*lac::phaC1AB*	Km^r^; *phaC1*_*Re*_; *phaA*_*Re*_; *phaB1*_*Re*_	This study
pBBR1MCS-2 ::p*lac::aldD::dhaT::pct* ::p*lac::phaC1AB*	Km^r^; *aldD*_*Pp*_; *dhaT*_*Pp*_; *pct*_*Cp*_; *phaC1*_*Re*_; *phaA*_*Re*_; *phaB1*_*Re*_	This study

### Growth of cells

250-mL Erlenmeyer flasks containing 50 mL MMB medium [3.56 g/L Na_2_PO_4_ • 2 H_2_O, 0.68 g/L KH_2_PO_4_, 0.63 g/L (NH4)_2_SO_4_, 2.47 g/L MgSO_4_ • 7 H_2_O, 1.0% (vol/vol) trace element solution (0.1 N HCl in 4.2 g/L FeSO_4_ • 7 H_2_O, 5.0 g/L CaCl_2_ • 2H_2_O, 2.4 g/L CoCl_2_ • 6 H_2_O, 0.58 g/L CuCl_2_ • 2 H_2_O, 2 mg/L NiCl_2_ • 6 H_2_O, 3 mg/L MnCl_2_ • 4 H_2_O, 0.03 g/L H_3_BO_3_, 4.3 g/L ZnSO_4_ • 7 H_2_O, 3 mg/L NaMoO_4_ • 2 H_2_O)] with 300 mM of glycerol was used for optimization of culture medium. Cells were cultivated in 250-mL Erlenmeyer flasks at an agitation of 125 rpm and at 30°C. High cell density fed-batch cultivation of *S. blattae* were conducted in a 2 L jar fermenter (Biostat B plus, Sartorius AG, Göttingen, Germany) containing 1.5 L of basal medium (BM) (Andreeßen et al. 2014a,b) or MMB medium with 300 mM of glycerol as carbon source.

Glycerol was intermittently added to the culture medium to maintain a concentration between 50 and 300 mM. 500-mL flasks containing 100 mL BM or MMB medium, 300 mM of glycerol and 50 μg/L of kanamycin were used for seed cultivations. Dissolved oxygen was monitored and pH was controlled in the range of 6.8 – 6.9 by using a 7.5% aqueous solution of ammonium hydroxide.

### Plasmid construction and transfer into *E. coli* and *S. blattae*

All processing and manipulation of DNA was carried out as described by Sambrook et al., 1989. Plasmid pBHR68 (Spiekermann et al., 1999) was digested with *Bsp*119I and *Eco*RI to generate a 4.2-kbp fragment comprising the coding regions of *phaC1*, *phaA* and *phaB1*. This fragment was ligated to the *Cla*I and *Eco*RI restriction fragment of pBBR1MCS-2 (Kovach et al., 1995) to generate pBBR1MCS-2::*p*_*lac*_::*phaC1AB*. Then, pBBR1MCS-2::*p*_*lac*_::*phaC1AB* was digested with *Ssp*I to generate a 4.7-kbp expression cassette of the *phaC1*, *phaA* and *phaB1* under control of the *lac* promoter and ligated with the *Eco*ICRI linearized fragment of pBBR1MCS-2::*aldD*::*dhaT*::*pct* (Heinrich et al., 2013) to generate the expression vector pBBR1MCS-2::*p*_*lac*_::*aldD*:*:dhaT*::*pct*::*p*_*lac*_::*phaC1AB*. In addition, *S. blattae* ATCC33430 was transformed with pBBR1MCS-2::*p*_*lac*_::*aldD*:*:dhaT*::*pct*::*p*_*lac*_::*phaC1AB* to generate *Sb*6BP by electroporation as previously described (Heinrich et al., 2013).

### Optimization of cultivation medium

When optimizing the medium, we thought yeast extract is not necessary, because cultivations in complete synthetic medium have been made for bacteria such as *Klebsiella pneumonia* (Brandl et al., 1998), *E. coli* (Enayati et al., 1999) or *R. eutropha* (Sato et al., 2013). Therefore, several different concentrations of yeast extract were tested as described below.

250-mL Erlenmeyer flasks containing 50 mL MMB medium with 0, 0.2 or 2.0 (g/L) of yeast extract, respectively were used to cultivate *S. blattae* ATCC33430. The optical density at 600 nm and the pH were measured in samples withdrawn from the culture. In order to decide which yeast extract concentration is favorable for high cell density cultivation, 2 L bioreactors containing 1.5 L of MMB medium with 300 mM of glycerol and 0.2, 0.67 or 6.7 g/L of yeast extract and *Sb*6BP were used. Cell densities (g_CDW_/L) and polymer contents (% wt_PHA_/wt_CDW_) were measured.

### Synthesis and purification of poly(3HB-*co*-3HP)

PHA was synthesized in a 2 L bioreactors containing 1.5 L of BM or MMB medium for 72 or 48 h. Glycerol was used as sole carbon source. Generally, enteric bacteria also *S. blattae* synthesize 1,3PD only under anaerobic condition. Thus, 4 different cultivation conditions (aerobic, anaerobic, low aeration/agitation and two-step) were conducted to optimize poly(3HB-co-3HP) synthesize condition in recombinant *S. blattae* (*SB*6P).

The operating conditions were as follows: Aerobic condition means an agitation at 800 rpm and an aeration rate of 2.0 L/min. Anaerobic conditions were maintained at an agitation of 150 rpm without any aeration whereas low aeration/agitation conditions were provided at a stirring rate of 150 rpm and an aeration rate of 0.4 L/min. 150 rpm was the minimum stirring rate required in medium mixing. The two-step fermentation (the first 24 hours under anaerobic condition, thereafter, aerobic condition) was performed according to Heinrich et al., 2013.

### Cell harvest and extraction of poly(3HB-*co*-3HP)

After separating the cells from the culture broth, cells were frozen at −30°C and freeze dried. Poly(3HB-*co*-3HP) or poly(3HB) was isolated from the pulverized dry cell matter by digestion of non-PHA biomass employing a 13% (vol/vol) sodium hypochlorite solution (Heinrich et al., 2013, Heinrich et al., 2012).

### Determination of poly(3HB-*co*-3HP)

**Figure 2:**
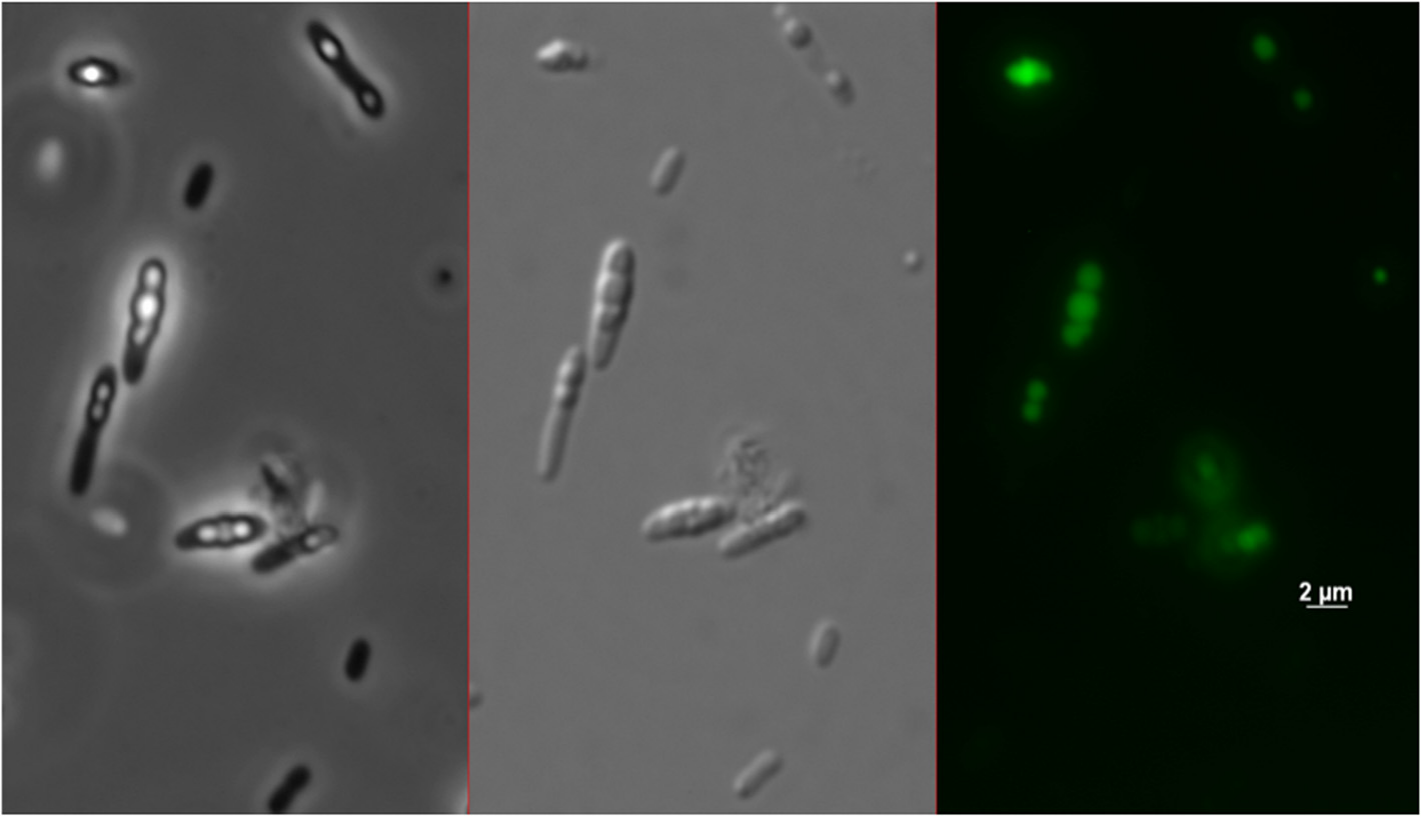
**Poly(3HB-** ***co*** **-3HP) accumulation of** ***Shimwellia blattae Sb*** **6BP.** After 72 h of fermentation in BM under two-step condition. Hydrophobic inclusions were stained with Nile red and observed with a fluorescence microscope employing phase contrast (left), differential interference contrast (middle), and fluorescence microscopy at 312 nm (right), respectively.

Analysis of polymer content and purity of the extracted polymer was done by gas chromatography (GC). For this dried cell mass or samples of isolated poly(3HB-*co*-3HP) and poly(3HB) were exposed to acidic methanolysis as described before (Brandl et al., 1998; Timm et al., 1990). For microscopic analysis of PHA-granules cells were stained with Nile red (Spiekermann et al., 1999) (Figure 2).

### Determination of glycerol and 1,3PD

Concentrations of glycerol and 1,3PD in the media were monitored by HPLC analysis. For this, supernatants were assayed using a Lachrom Elite HPLC-System (VWR-Hitachi, Darmstadt, D) chromatograph with a RI-detector (Type 2490 VWR, Darmstadt, D) and a Metacarb 67H-column (300 × 6.5 mM, VWR-Varian, Darmstadt, D) at 75°C and at a flow rate of 0.8 ml/min for 20 min. The mobile phase was 4.5 mM sulfuric acid.

## Results

### Synthesis of poly(3HB-*co*-3HP) influenced by cultivation conditions

In order to develop a process for poly(3HB-*co*-3HP) production by a newly engineered *Sb*6BP strain, cultivation was conducted under four different conditions (aerobic, two-step, low aeration/agitation and aerobic conditions). (i) Aerobic condition occurred at high aeration and agitation, (ii) the two-step condition occurred during the first 24 hours under anaerobic condition and thereafter under aerobic condition, (iii) for low aeration/agitation condition a minimum stirring rate was applied together with low rate of aeration and (iv) for anaerobic condition was a minimum stirring rate was applied without aeration.

**Figure 3:**
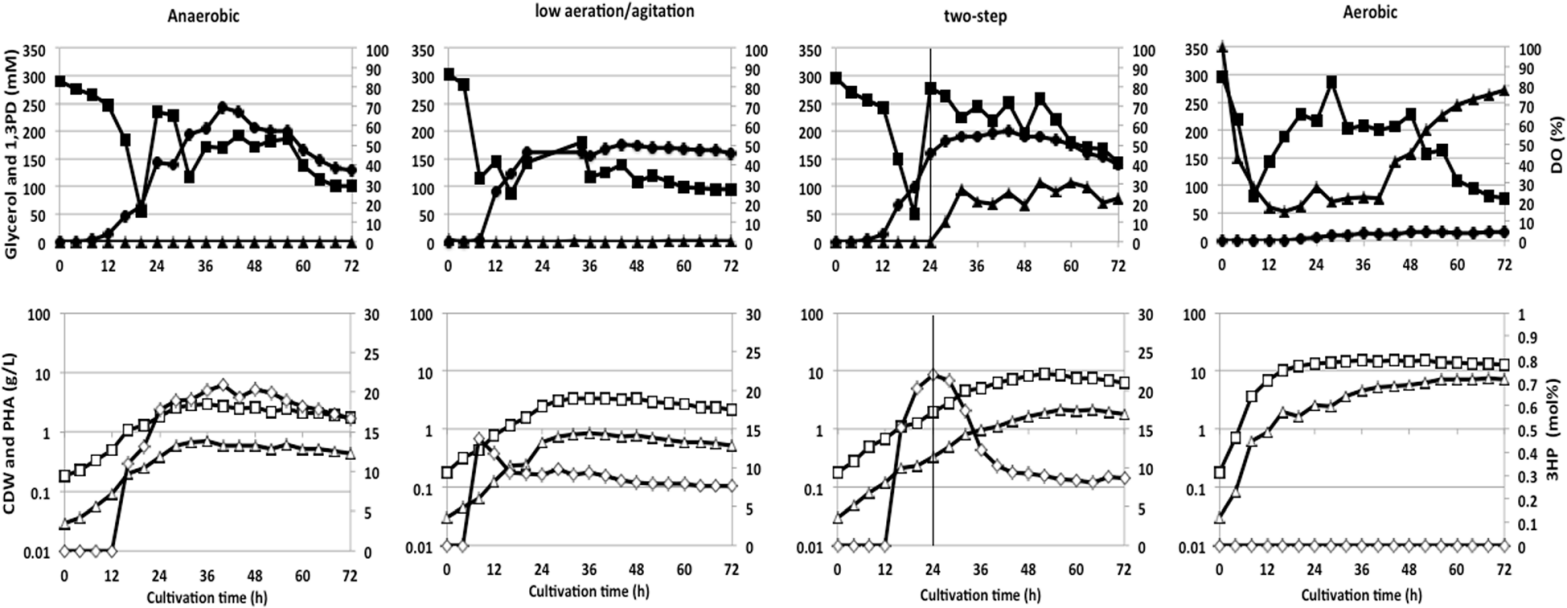
**Fed-batch profile of Shimwellia blattae Sb6BP under different cultivation conditions in BM.** The cultures were grown in 2 L jar-fermenter at 30°C. 1,3PD (●), glycerol (■), dissolved oxygen (▲), CDW (□), poly(3HB-co-3HP) or poly(3HB) (△), 3HP (◇).

**Figure 4:**
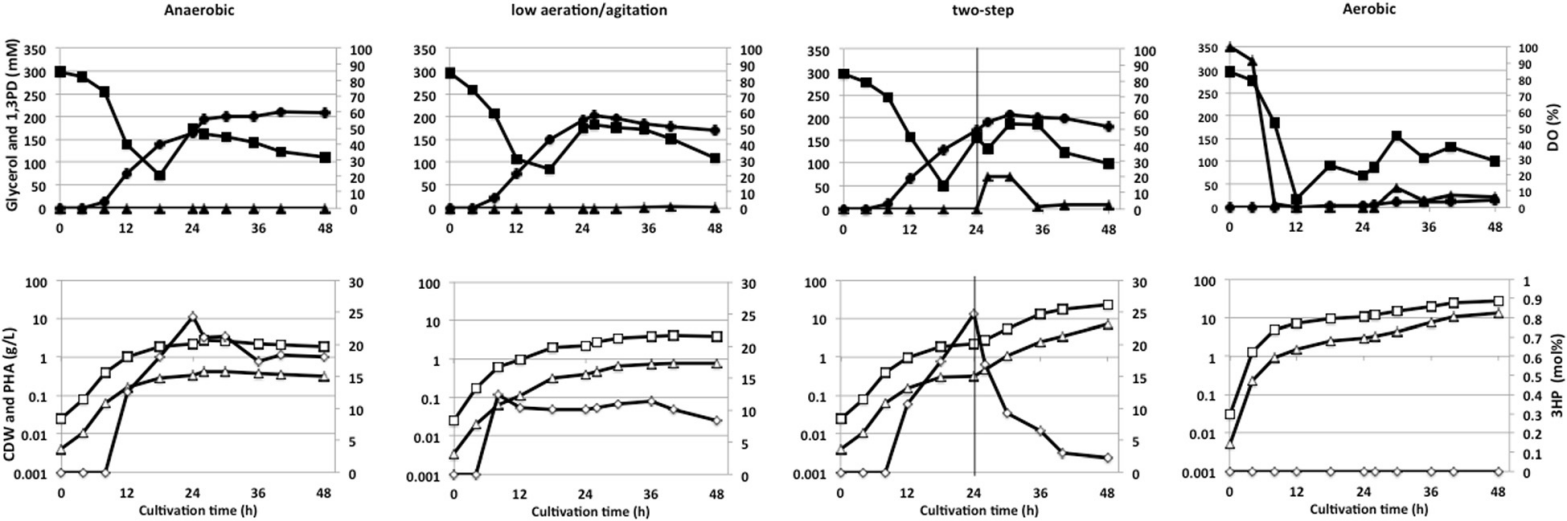
**Fed-batch profile of Shimwellia blattae Sb6BP under different cultivation conditions in MMB containing 0.67 g/L of yeast extract.** The cultures were grown in 2 L jar-fermenter at 30°C. 1,3PD (●), glycerol (■), dissolved oxygen (▲), CDW (□), poly(3HB-co-3HP) or poly(3HB) (△), 3HP (◇).

Glycerol supplementation started from 8 to 16 hours of cultivation in every culture when glycerol concentration became lower than 100 mM. In most cases, glycerol consumption rate became lower than supplementation rate after 18 h cultivation. If this was the case, the supplementation rate was regulated to maintain a glycerol concentration between 50 and 300 mM during the cultivations. In case of 1,3PD, it might not be effectively transported into the cell after it was transiently formed and excreted into the medium before it could be incorporated into the polymer via 3-hydroxypropionyl-CoA; thus the 1,3PD concentration was not so affected during cultivations. Moreover, the reduction of 1,3PD concentration in the later phase of the cultivation period could also occur due to a dilution effect (Figures 3 and 4).

The amount of 1,3PD that was converted into 3-hydroxypropionyl-CoA and polymerized into poly(3HB-*co*-3HP) was too small to affect the 1,3PD concentration of the culture medium in these studies. We already confirmed that 1,3PD supplemented to the culture medium was converted to 3-hydroxypropiony-CoA which is polymerized into poly(3HB-*co*-3HP) in the recombinant strain in which *dhaT* was heterologously expressed. However, efficiency was very low (data not shown). Therefore, most of the 3HP monomer in the accumulated polymer was provided directly from 3-hydroxypropionaldehyde via 3-hydroxypropionate and 3-hydroxyproionyl-CoA by AldD and Pct, respectively.

Strain *Sb*6BP was confirmed as a poly(3HB-*co*-3HP) producer (Table 2). Moreover, strain *Sb*6BP synthesized the poly(3HB-*co*-3HP) copolymer only under anaerobic, two-step and low aeration/agitation conditions. Under aerobic condition no 3HP were incorporated as constituent. The productivity for poly(3HB-*co*-3HP) in BM medium ranged from 4 mg_PHA_/L/h to 25 mg_PHA_/L/h and was highest under the two-step condition. No copolymer but poly(3HB) homopolymer was formed under aerobic conditions, and the productivity for poly(3HB) was higher in comparison to poly(3HB-*co*-3HP) in both BM and MMB medium. The polymer content was also highest in the two-step fermentations (29.9% in BM and 30.7% wt_PHA_/wt_CDW_ in MMB, respectively), and lowest (14.7% in BM and 17.2% wt_PHA_/wt_CDW_ in MMB, respectively) in the anaerobic fermentation. On the other hand, poly(3HB-*co*-3HP) with the highest 3HP content was obtained (16.6 mol% in BM and 18.1 mol% in MMB, respectively) in anaerobic fermentations.

**Table 2:** **Results of fed-batch cultivation using** ***Sb*** **6BP and glycerol as sole carbon source**

**Strain**	**Medium**	**Condition**	**Cell density (g**_**CDW**_ **/L)**	**PHA content (wt%)**	**PHA (g**_**PHA**_ **/L)**	**Monomer composition (mol%)**		**Cultivation time (h)**	**PHB(P) productivity (mg**_**PHA**_ **/L/h)**	**Glycerol yield**
						**3HB**	**3HP**			
*Sb*6BP	BM	Anaerobic	1.72	14.7	0.25	83.4	16.6	72	3.51	0.0041
		Low aeration/agitation	2.11	24.6	0.52	92.4	7.3		7.21	0.0075
		Two-step	6.12	29.9	1.83	91.4	8.6		25.4	0.032
		Aerobic	12.6	55.2	6.96	100	0		96.6	0.097
	MMB	Anaerobic	1.84	17.2	0.32	82.9	18.1	48	6.59	0.0033
		Low aeration/agitation	3.78	20.3	0.77	91.7	8.3		16.0	0.011
		Two-step	23.2	30.7	7.12	97.9	2.1		148.4	0.093
		Aerobic	27.3	48.1	13.1	100	0		273.6	0.15

Cells were cultivated in MB or MMB medium with glycerol that was intermittently added. MMB contained 0.67 g/L yeast extract. Kanamycin was added as 50 μg/ml final concentration at the beginning of the cultivation. All cultivations were conducted in a 2 L jar-fermenter.

The time courses of polymer synthesis for each cultivation condition in BM medium were as follows (Figure 3): (1) Anaerobic: cell growth and addition of ammonium hydroxide almost stopped after 48 h cultivation time. The highest 3HP composition (20.2 mol%), 1,3PD concentration (242 mM) and polymer production was recorded after about 40 h cultivation time. These data indicate that 3HP-CoA and 1,3PD synthesis occurred only during exponential cell growth. Moreover, it was indicated that both, (*R*)-3HB-CoA and 3HP-CoA are not supplied after the stop of cell growth, because the 3HP content was very stable after 40 h. (2) Low aeration/agitation: the time courses of every parameter except 3HP monomer fraction were very similar when compared to anaerobic conditions as explained above. The highest molar 3HP monomer fraction was recorded earlier than 12 hour of culture time. The residual cell mass was only marginally influenced by aeration, but the 3HP monomer content of the polyester was decreased to less than 50% in comparison to anaerobic condition. (3) Two-step: Interestingly, the highest molar 3HP fraction in the copolymer was recorded at the time when the cultivation conditions were just switched. After that, the fraction of 3HP moieties rapidly dropped but the cell dry weight (CDW) increased on the other hand. Formation of 1,3PD was maintained for some hours after the cultivation condition was changed from anaerobic to aerobic and then stopped. (4) Aerobic: only the poly(3HB) homopolymer was synthesized under aerobic conditions. Furthermore, the cell density (12.6 g_CDW_/L) and the PHA productivity (96.6 mg_PHA_/L/h) were the highest. Only a small amount of 1,3PD (10 to 15 mM) was synthesized after 24 h of cultivation, and the 3HP monomer was not detected by GC analysis at any period.

GPC analysis of the isolated poly(3HB-*co*-3HP) obtained in BM after two-step cultivation for 72 h revealed an average molecular weight of 765,293 Da with a polydispersity index (M_w_/M_n_) of 2.49.

### Optimization of the medium for cultivation

Although cells of strain *Sb*6BP could be successfully cultivated, poly(3HB-*co*-3HP) productivity was still low due to a low residual cell mass. The cell density was only 12.6 g_CDW_/L under aerobic condition in BM medium. From these results it was suspected that some substances required for cell growth were missing or that at least a severe shortage had occurred in BM medium. Therefore, the medium was optimized.

**Figure 5:**
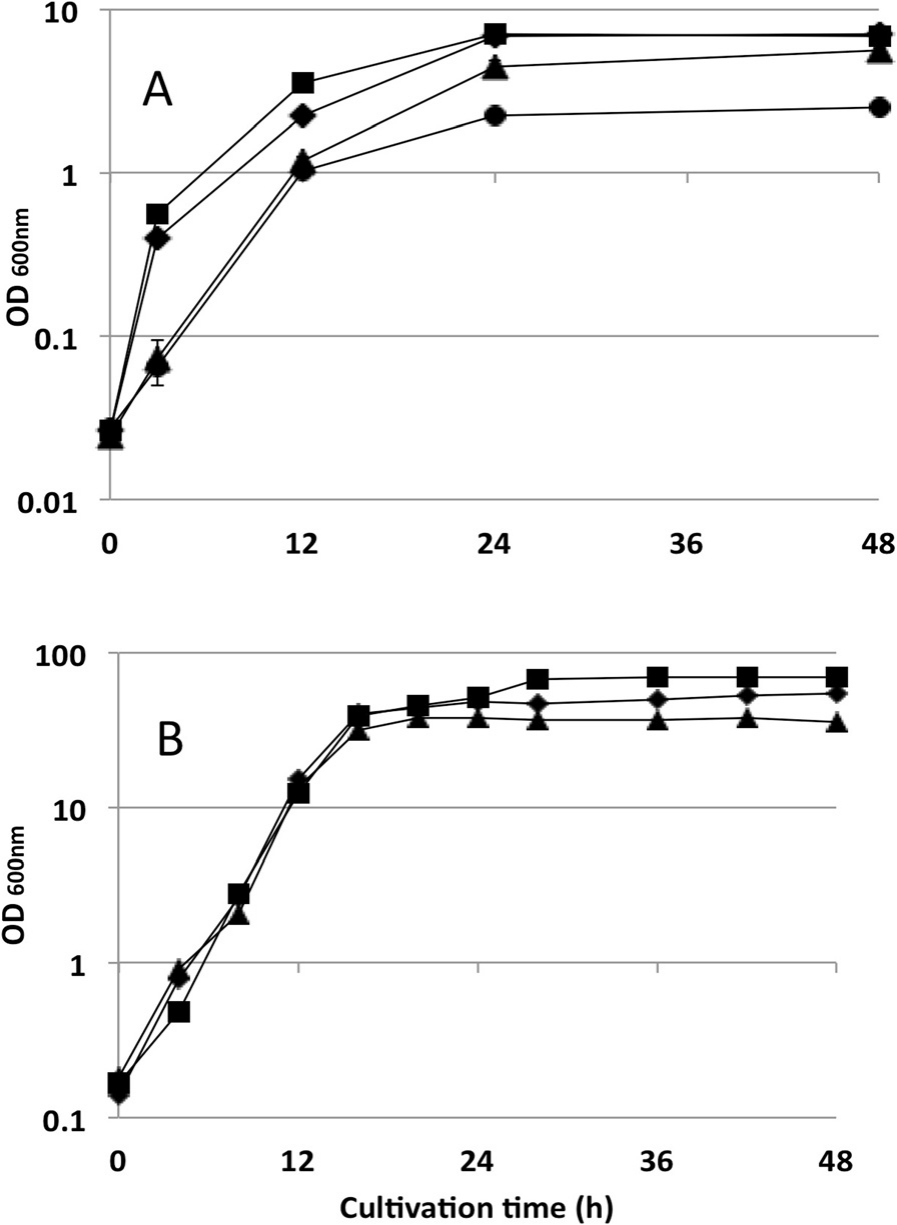
**Cultivation of Shimwellia blattae Sb6BP under aerobic condition in flask (A) and 2 L jar-fermenter (B) in MMB with various yeast extract concentration and 300 mM glycerol. (A)**: yeast extract concentration are 2.0 (■), 0.2 (◆), 0 (▲) and 0 g/L (●), respectively. Trace element was not added to a case of (●). **(B)**: yeast extract concentration are 6.7 (■), 0.67 (◆), 0.2 g/L (▲), respectively.

It was confirmed that the growth rate of the cells was influenced by the concentration of yeast extract in the medium (Figure 5). The growth rates (μ) of the cells in MMB between 0 to 12 h cultivation time containing 0, 0.2 or 2.0 g/L of yeast extract were 0.32, 0.36 and 0.41 h^−1^, respectively. It was also confirmed that the trace element solution used in this study is necessary for cell growth (Figure 5A). These results indicate in contrast to our expectations that yeast extract is very important for cell growth of *S. blattae*. Yeast extract contains many vitamins like biotin, 4-aminobenzoic acid, pantothenic acid, pyridoxine, riboflavin and thiamine (data from DIFCO) in addition to amino acids and some of these compounds are likely to be growth limiting.

Although yeast extract was confirmed as a key factor for growth of *S. blattae*, the optimum concentration could not be identified in flask culture experiments since cell growth stopped after 24 h of cultivation time when the pH had dropped below 5. Therefore, higher cell density cultivations were conducted in 2 L fermenter in order to determine the optimum concentration of yeast extract. The cell densities obtained in aerobic cultivations in MMB containing 0.2, 0.67 and 6.7 g/L of yeast extract were 14.6, 27.3 and 44.6 g_CDW_/L, respectively (Figure 5B, Table 3).

**Table 3:** **Cultivation of** ***Sb*** **6BP in MMB containing various concentrations of yeast extract**

**Yeast extract (g/L)**	**Cell density (g**_**CDW**_ **/L)**	**PHA (g**_**PHA**_ **/L)**	**PHA content (wt%)**	**Cultivation time (h)**
0.2	14.6	5.58	38.3	48
0.67	27.3	14.2	48.1	48
6.7	44.6	21.1	47.2	48

During cultivation, glycerol was intermittently added. Kanamycin was added as 50 μg/ml final concentration at the beginning of the cultivation. All cultivations were conducted in a 2 L jar-fermenter.

With regard to the cost, we chose of 0.67 g/L to obtain 27.3 g_CDW_/L.

### Synthesis of poly(3HB-*co*-3HP) in MMB medium

Improved productivities for PHA synthesis were between 1.9 and 5.8 times higher in MMB than in BM; the highest poly(3HB-*co*-3HP) productivity was obtained in two-step cultivation (25 mg_PHA_/L/h in BM medium in comparison to 148 mg_PHA_/L/h in MMB) (Table 2). The positive effect of MMB medium was highest in two-step cultivation experiments. This is explained by the fact that cell growth was very high after the culture conditions were changed (Figure 4).

Conversely, these effects were less under anaerobic and low aeration/agitation conditions. An explanation is most probably because the rate-limiting factor was oxygen and not one of the compounds included in yeast extract. These observations could not be confirmed in MMB under aerobic condition as there was no poly(3HB-*co*-3HP) accumulated. The polymer content and the 3HP fraction in the copolyester were not so much influenced under anaerobic and low aeration/agitation conditions whereas the 3HP fraction in two-step condition dropped rapidly from 8.6 to 2.1 mol% after the cultivation conditions were changed (Figure 4). These results indicate that the decrease of 3HP fraction might be due to a relative decrease of the monomer composition by additional accumulation of 3HB and/or poly(3HB), because, after switching the conditions, cell growth started again and 3HB-CoA provision via acetoacetyl-CoA was started as well. In addition, it was confirmed that the net 3HP amount increased until 30 h; thereafter no increase occurred in case of two-step cultivation when using the MMB medium. Therefore, there is a possibility that the resulting polymer was likely a blend of poly(3HB) and poly(3HB-*co*-3HP).

Poly(3HB-*co*-3HP) with the highest 3HP fraction (18.1 mol%) was obtained during anaerobic condition; however, the polymer productivity and cell density were only 6.6 mg_PHA_/L/h and 1.84 g_CDW_/L, respectively.

## Discussion

In this study we engineered a recombinant strain of *S. blattae* ATCC33430 (*Sb*6BP), which is able to synthesize poly(3HB) and poly(3HB-*co*-3HP). However, *Sb*6BP synthesized the copolymer only under oxygen limiting conditions because the cells produced only small amounts of 1,3-propanediol (1,3PD) as a precursor of 3-hydroxypropionyl-CoA (3HP-CoA) under aerobic condition. In any case, in comparison with the 1,3PD productivity under oxygen limiting conditions, a lower productivity of 1,3PD under aerobic condition causes non-accumulation of poly(3HB-*co*-3HP).

However, successful production of poly(3HB-*co*-3HP) in recombinant *S. blattae* was achieved, 3HP composition was rather low. The glass transition temperature of poly(3HB-*co*-3HP) decreases rapidly from −3°C to −15°C, and the melting temperature (Tm) decreases from 163°C to 73°C as the 3HP fraction in the copolymer increased from 25.6 to 36.3 mol% (Wang et al., 2013). In particular, the poly(3HB-*co*-3HP) with a 3HP content exceeding 30 mol% is therefore much more flexible and less crystalline and is expected to approach that of conventional plastics such as polypropylene, polystyrene, and polyethylene. The low 3HP fraction indicates that the strain cannot actively import 1,3PD from culture medium or that 3HP-CoA supply via 3-hydroxypropionaldehyde and 3-hydroxypropionate is insufficient.

This is the first report for the production of poly(3HB-*co*-3HP) without using vitamin B_12_ and expensive compounds such as 3-hydroxypropionate by recombinant *S. blattae*. We achieved a poly(3HB-*co*-3HP) productivity of 148 mg_PHA_/L/h in this study, which is the highest so far reported (Shimamura et al., 1994; Fukui et al., 2009; Wang and Inoue, 2001; Wang et al., 2013). *S. blattae* is therefore one of the promising bacterial strains for poly(3HB-*co*-3HP) production from glycerol.

However, the reported processes still require oxygen limitation. Therefore, the polymer productivity is still very low. In addition, the polymer produced by two-step cultivation method was likely a blend of poly(3HB) and poly(3HB-*co*-3HP) owing to the conditional change from anaerobic to aerobic condition. The remaining challenges are to achieve efficient utilization of 3HPA or 1,3PD and the expression of the genes regulated by *dha* regulon in aerobic conditions for aerobic production of poly(3HB-*co*-3HP). Moreover, an enhancement of the metabolite flow from 3-hydroxypropionaldehyde to 3HP-CoA will be necessary to increase the 3HP fraction in the copolymer. By using the *pduP* gene from *Salmonella enterica* (Andreeßen et al., 2010) or *Salmonella typhimurium* (Gao et al., 2014), the provision of 3HP-CoA provision might be improved.
